# GDF-15/GFRAL Axis Dynamics and Vulnerability Windows in Bariatric Surgery: Pathophysiological, Microbiomic, and Pharmacological Perspectives

**DOI:** 10.3390/cells15191823

**Published:** 2026-10-07

**Authors:** Alin Constantin Pînzariu, Ionela Lăcrămioara Șerban, Radu Petru Soroceanu, Vlad Ionuț Vlăsceanu, Madalina Maxim, Ancuta Andreea Miler, Sebastian Marian Leonte, Carmen Atodiresei, Mihaela Moscalu, Lorena Mihaela Manole, Laura Mihaela Trandafir, Dragomir Nicolae Șerban, Daniel Vasile Timofte

**Affiliations:** 1 Grigore T. Popa University of Medicine and Pharmacy Iasi, 700115 Iasi, Romania; alin.pinzariu@umfiasi.ro (A.C.P.); ionela.serban@umfiasi.ro (I.L.Ș.); petru.soroceanu@umfiasi.ro (R.P.S.); vlasceanu.vlad@yahoo.com (V.I.V.); mg-rom-33565@students.umfiasi.ro (S.M.L.); carmen.atodiresei@d.umfiasi.ro (C.A.); mihaela.moscalu@umfiasi.ro (M.M.); lorena.manole@umfiasi.ro (L.M.M.); laura.trandafir@umfiasi.ro (L.M.T.); dragomir.serban@umfiasi.ro (D.N.Ș.); daniel.timofte@umfiasi.ro (D.V.T.); 2 Department of Surgery, Sf. Spiridon County Emergency Clinical Hospital, 700111 Iasi, Romania; 3 Institute of Gastroenterology and Hepatology, Sf. Spiridon County Emergency Clinical Hospital, 700111 Iasi, Romania; 4 Sfânta Maria Emergency Children Hospital, 700309 Iași, Romania

**Keywords:** bariatric surgery, GDF-15/GFRAL/RET axis, GLP-1-based pharmacotherapy, food aversion, gut microbiota, SCFAs, gut–brain axis, MT1-MMP, semaglutide, incretin-based therapies

## Abstract

Bariatric surgery (BS) and new pharmacological treatments cause significant neurometabolic changes. However, the pathways linking peripheral stress to central metabolic control remain poorly understood. This review highlights the GDF-15/GFRAL/RET complex as a key integrator of multiorgan-to-brain stress signals that shape systemic metabolic adaptation after surgical and pharmacological interventions. Based on a structured search across PubMed, Embase, Scopus, Google Scholar, and Web of Science covering 2020–2026 alongside foundational studies, we integrated translational evidence from peer-reviewed human trials and mechanistic animal models to map the GDF-15/GFRAL/RET axis across bariatric and incretin domains. We examined the neurobiology of the GDF-15/GFRAL axis to clarify variability in metabolic and behavioral outcomes after BS. This axis links metabolic stress signals from the gut, liver, and adipose tissue to the hindbrain and hypothalamic regulation of energy balance. Changes in the gut microbiome and SCFA–FFAR2/FFAR3 signaling after surgery interact with GDF-15 and incretin pathways. Future studies should critically compare BS with synergistic or alternative non-invasive neurometabolic approaches such as incretin mimetics, exogenous GDF-15 therapies, and high-protein diets.

## 1. Introduction

Obesity is a complex, chronic systemic disease involving metabolic and neurobehavioral dysfunctions and a major contributor to severe comorbidities [1].

While intensive diet and exercise programs typically result in only 5–10% weight loss that is often not sustained over time [2,3], GLP-1 receptor agonists and BS function as complementary interventions that alter the defended body weight set point through neuroendocrine, metabolic, and inflammatory adaptations [4,5,6,7]. Surgical intervention reshapes gut-derived signaling networks and stress-responsive cytokine dynamics, delivering both weight-dependent and organ-specific, weight-independent therapeutic outcomes [5,6,7].

Targeted perioperative nutritional strategies may use growth differentiation factor 15 (GDF-15), a stress-induced cytokine with diverse biological functions and a member of the transforming growth factor beta (TGF-β) superfamily [2,8,9,10], as a neurometabolic ligand before and after surgery.

Despite these metabolic benefits, a complex neuropsychiatric phenotype remains after BS [11] that is often overlooked. A subgroup of post-bariatric patients experiences paradoxical neuropsychiatric decompensation [12,13]. While the postoperative reduction in appetite and related behavioral changes have been interpreted as passive responses to surgical trauma, these changes may reflect active affective–cognitive adaptations regulated by neuroimmune circuits and peripheral inflammatory signaling [12].

These adaptations unfold across what we term perioperative vulnerability windows: bounded postoperative intervals in which intense GDF-15/GFRAL flux coincides with heightened nutritional risk and altered neurobehavioral coping so that the same signal supporting adaptive energy restriction may instead produce maladaptive aversion, nutritional deficit or behavioral decompensation. The construct is temporal, biological, nutritional and behavioral at once and is proposed as an organizing framework rather than a validated clinical classification.

Although a direct link between persistent GDF-15 elevation, visceral discomfort or aversion, and psychiatric symptoms after bariatric intervention has not been established [14,15,16,17], we hypothesize that the targeted nutritional modulation of the GDF-15/GFRAL/RET axis may protect the dopaminergic reward circuit over time and help prevent anhedonia and addiction transfer. This supports a translational approach to using GDF-15 to support the post-bariatric transition to efficient neurometabolic adaptation without triggering emetic or psychiatric decompensation.

## 2. Materials and Methods

This article is a narrative, integrative review. It did not follow PRISMA or another systematic review reporting framework, and it did not include a formal risk-of-bias assessment, record-level screening count, or quantitative synthesis. The procedures described below are reported to make the evidence base traceable, not to claim systematic completeness. Evidence was prioritized by proximity to the perioperative bariatric context and by study design, with human clinical data reported as the primary finding and preclinical data as mechanistic context; this distinction is made explicit in the tables and in Section 5.2 and Section 6.

### 2.1. Search Strategy and Literature Sources

We conducted a comprehensive literature search across PubMed/MEDLINE, Embase, Scopus, Web of Science, and Google Scholar, covering publications from 2020 to 2026, and selectively incorporated older studies to provide foundational historical context. The search strategy aimed to capture translational, pathophysiological, and clinical evidence on the GDF-15/GFRAL/RET signaling axis and its metabolic implications in BS, rather than to perform an exhaustive quantitative meta-analysis. Advanced Boolean logic (AND, OR) was applied to combine Medical Subject Headings (MeSH) and free-text title/abstract keywords. Core search terms included GDF-15, growth differentiation factor 15, GFRAL, RET signaling, BS, MT1-MMP/MMP14-mediated GFRAL cleavage, the integrated stress response (ISR), post-bariatric hypoglycemia, and concurrent incretin therapies, including GLP-1 receptor agonists.

### 2.2. Eligibility Criteria and Evidence Synthesis

Literature selection followed a screening protocol that assessed titles, abstracts, and full texts to identify peer-reviewed primary human clinical trials, mechanistic animal models, and relevant clinical studies published in English. We also reviewed secondary publications, such as narrative reviews, systematic reviews, editorials, commentaries, case series, and case reports, to provide context for emerging concepts. Book chapters, conference abstracts, and dissertations were excluded. Included studies were categorized and synthesized within core thematic domains related to peripheral metabolic stress and central neuroplasticity. Unless stated otherwise, “bariatric surgery” in this review refers to Roux-en-Y gastric bypass and sleeve gastrectomy, the two procedures for which perioperative GDF-15 data exist; the conclusions should not be extrapolated to other bariatric procedures.

## 3. The Fundamental Neurobiology of the GDF-15/GFRAL Axis and Its Role in the Heterogeneity of Postoperative Response in Bariatric Patients

### 3.1. Hindbrain-Restricted GFRAL Signaling and Satiety Circuits

GDF-15 acts as a systemic endocrine messenger released in response to various cellular and somatic stresses. It crosses the permeable blood–brain barrier and binds the GFRAL-RET receptor complex in the brainstem regions of the area postrema (AP) and the nucleus of the solitary tract (NTS) [18,19,20]. Although the GFRAL receptor is localized in the hindbrain, the GDF-15 activation of these neurons subsequently activates neurons in the parabrachial nucleus (PBN), initiating a neuronal circuit involving the central amygdala (CeA) and the cortex that regulates and reduces food intake [7,9,12].

GDF-15 promotes lipolysis through a communication circuit between the brain and somatic tissues, mediated by the GFRAL–β-adrenergic signaling axis and the stimulation of adipose triglyceride lipase expression [6,12]. Feetham et al. [21] suggest that the chemogenetic activation of brainstem neurons reduces food intake and stimulates fatty acid oxidation independently of visceral aversive circuits, while more intense stimulation causes energy expenditure and body temperature to plunge, progressing toward a state mimicking metabolic torpor.

The defining experimental evidence obtained from multispecies preclinical models and dedicated bariatric studies addressing the neuroanatomical architecture and translational dynamics of the GDF-15/GFRAL axis is synthesized in Table 1. The GFRAL–RET receptor complex is selectively expressed on glutamatergic neuronal populations, specifically CCK+ and SLC6A2+ subpopulations. Increasing doses of GDF-15 recruit ascending circuits to the lPBN, CeA, and PVH. This triggers integrated aversive responses and changes in gastric motility. Single-cell analyses reveal direct colocalization between GFRAL and *GLP1R* receptors in excitatory neuronal clusters within the AP. This provides the neurobiological basis for the synergistic action of GDF-15 and GLP-1 after surgical interventions. In vivo validation in bariatric murine models suggests that both RYGB and SG induce a sustained increase in plasma and tissue GDF-15 for up to 8 weeks. GDF-15 is predominantly expressed in the remnant stomach and ileum, shifting the discussion away from the hypothesis of a simple acute phenomenon caused by surgical stress.

Bariatric surgery induces rapid changes in enteroendocrine hormone secretion, an increase in endogenous GLP-1 and an increase in peptide tyrosine-tyrosine (PYY). Central neuroendocrine pathways orchestrate energy balance by employing complementary receptor dynamics. Brainstem amylin receptor signaling reduces food intake, while the activation of central Y2R receptors promotes satiety, as shown by subcutaneously administered PYY analogs such as peptide Y-14, which appeared to reduce food consumption compared with placebo [3]. At the hypothalamic level, GLP-1 activates POMC neurons and inhibits AgRP neurons via *GLP1R*, whereas PYY3–36 curtails food intake by modulating hypothalamic NPY and POMC neuron activity. Concurrently, CCK acts in the hindbrain via CCK1 receptors to induce satiety, whereas reduced ghrelin attenuates meal initiation signals by decreasing the stimulation of GHS-R1a receptors on arcuate nucleus neurons [25].

Sedentary behavior worsens metabolic dysfunction and low-grade systemic inflammation by disrupting neuroendocrine GLP-1 and GDF-15/GFRAL/RET axes and promoting gut dysbiosis. During chronic inactivity, the sustained pathological activation of the integrated stress response (ISR/ATF4) raises basal GDF-15 secretion, speeding up the desensitization of central GFRAL receptors and causing GDF-15 resistance with impaired satiety signaling.

Elucidating this complex neuroendocrine landscape can help decode inter-individual variability in postoperative bariatric trajectories. Surgical interventions reshape these overlapping peptidergic pathways, creating a dynamic post-procedural state where GDF-15 signaling works with GLP-1, PYY, and CCK while overriding ghrelin-driven hunger signals. Pre-surgical receptor desensitization, combined with individual differences in central multi-receptor responsiveness, underpins the marked inter-patient heterogeneity seen in postoperative satiety dynamics, visceral aversion tolerance, and long-term metabolic outcomes. Early postoperative adaptations, together with alterations in gut–brain axis signaling and bile acid pathways, may contribute to establishing a new defended weight set point, although in the long term, gradual weight regain is observed in a subgroup of patients [26].

The consequences of pathway activation are context-dependent, and four exposure settings should not be conflated. Physiological concentrations fluctuate within a narrow range and do not track meals or acute nutritional status, and physiological rather than pharmacological exposure does not suppress feeding. Disease-associated elevation, as in cancer, infection and chronic organ failure, accompanies anorexia and sickness behavior. Pharmacological exposure achieved with recombinant ligands or receptor agonists reproduces anorexia but also reproduces nausea and emesis at the doses required for weight loss. Elevation after bariatric surgery is intermediate in magnitude and the least characterized of the four. GFRAL expression outside the area postrema and the nucleus of the solitary tract has been reported but remains inconsistent across studies; the anorectic and aversive effects described here are attributable to the hindbrain population.

### 3.2. GDF-15 Kinetic Profiles in RYGB and SG

Although plasma GDF-15 levels are already high in patients with obesity, after bariatric surgery via RYGB, they increase even further at one year postoperatively, despite weight loss. This postoperative increase is not associated with cachexia, being considered a physiological compensatory mechanism to limit energy intake and maintain reduced weight [27,28,29].

Di Vincenzo et al. [30] show that SG increases plasma GDF-15 levels only in patients with obesity who do not have metabolic syndrome, while the presence of metabolic syndrome attenuates this effect of the surgical intervention, despite the fact that these patients initially have higher concentrations of this hormone.

There are divergent GDF-15 variations following bariatric interventions. Particularly after RYGB or SG, a postoperative increase in circulating GDF-15 levels is reported, which correlates positively with the magnitude of weight loss.

The one-year post-intervention results indicate a pronounced drop in this cytokine, mirroring weight loss. These discrepancies may be attributed to differences in baseline GDF-15 levels, age differences among subjects, small study sample sizes, the specific type of surgical procedure used, and individual physiological variability regarding the cytokine’s secretion profile [6,9,31]. Salmón-Gómez et al. [32] support that post-bypass GDF-15 variations are transient and time-limited, while Urones et al. [7] foreground an inverse correlation between preoperative GDF-15 and body fat percentage in patients with obesity.

Table 2 presents evidence from human studies comparing the temporal patterns and effects of circulating GDF-15 following RYGB and SG, integrating diverse study designs, sampling methods, serum kinetics, metabolic outcomes, and analytical limitations to clarify procedure-specific endocrine dynamics.

The evaluated studies have substantial methodological constraints. Numerous cohorts rely on single-center, non-randomized, or cross-sectional designs that lack unoperated control groups, paired intra-individual baselines, or critical early-phase sampling windows.

Marked analytical heterogeneity complicates cross-study comparisons, driven by divergent immunoassay platforms and non-standardized sample handling and storage conditions. Metabolic confounders are frequently undercontrolled, including unadjusted antidiabetic regimens, unmonitored dietary intake, and baseline imbalances in age, BMI, or glycemic status across surgical arms.

No study reported GDF-15 concentrations within the first 72 h after surgery. The earliest documented measurement was at 1 month [33]. Schmid et al. [36] collected samples 3–4 days after RYGB but did not report GDF-15 levels for that period. All values are in pg/mL, primarily expressed as the mean ± SD. Martinussen and Urones reported SEM, while Chaiyasoot reported median values. All studies used ELISA except Chaiyasoot, who used a multiplex assay. Baseline GDF-15 levels ranged from approximately 215 to 1165 pg/mL, which limits direct comparability between studies.

As the available evidence is derived mainly from small, single-center cohorts with limited ethnic diversity, these findings cannot be broadly generalized to global bariatric populations. Establishing definitive, population-level GDF-15 kinetic profiles will require prospective, multicenter trials employing standardized assay platforms, the rigorous control of metabolic co-medications, and harmonized longitudinal sampling protocols.

Richter et al. [37] found that GDF-15 levels are generally unaffected by changes in macronutrient composition or by a 6% weight loss alone. In the context of the extreme energy deficit induced by bariatric surgery, managing energy intake is closely linked to the ability to induce early satiety and regulate hunger mechanisms.

Clinical data support a correlation between the protein density of the post-bariatric diet and the subjective perception of satiety. Patients with stable weight at long-term follow-up after bariatric surgery achieve a higher daily protein intake, of approximately 1.2 ± 0.2 g/kg/day, compared to those facing weight regain, where intake decreases to 1.0 ± 0.2 g/kg/day, the latter having a higher consumption of carbohydrates [38].

To our knowledge, the literature shows that GDF-15 is unaffected by changes in macronutrient composition or high-protein diets [37], and plasmatic GDF-15 levels do not respond to food ingestion [34]. A modest 18% increase in GDF-15 was observed after weight loss associated with a carbohydrate-reduced high-protein (CRHP) diet but exclusively in patients treated concomitantly with metformin, suggesting that pharmacological interaction, not macronutrients, mediates this biomarker [37].

The kinetic evidence above delineates four such windows. No human study has reported GDF-15 concentrations within the acute window (0–72 h), which is therefore an evidence gap rather than a characterized phase.

The early adaptive window (1–3 months) carries the highest reported concentrations after SG and coincides with the transition to solid food, the lowest food tolerance and the steepest loss of fat-free mass [39,40].

In the intermediate window (3–12 months), procedures and phenotypes diverge: elevation persists in some SG cohorts, declines beyond 8 months after gastric bypass, and is attenuated from the outset in patients with preoperative metabolic syndrome [30,32]. In the late window (beyond 12 months), concentrations approach control values [34], coinciding in a subgroup with weight regain and reduced protein intake. The nutritional and neurobehavioral consequences of each window, and their potential use as intervention points, are addressed in Section 5.3 and Section 6.

### 3.3. Neurometabolic Integration and Aversive Behaviors

The GDF-15/GFRAL axis shares anatomical and functional pathways with neural circuits responsible for emesis, conditioned taste aversion (CTA), and disease-induced anorexia, suggesting that post-surgical metabolic adaptation depends on the interaction between homeostatic appetite regulation and aversive conditioning. The activation of these circuits controls appetite, food intake, and energy homeostasis while also being capable of triggering aversive behaviors, such as the sensation of nausea and vomiting [7,9].

In preclinical models, non-hypothalamic anorexia and weight loss are attributed to visceral malaise mediated by GFRAL receptors in the AP/NTS accompanied by pica and emesis, with weight loss driven primarily by visceral malaise via CGRP neuron activation in the PBN [13,41], whereas improvement in insulin sensitivity is secondarily mediated by caloric restriction and pair-feeding [13,32].

In humans, the response varies by surgical procedure. RYGB patients show a transient acute GDF-15 elevation (0–8 months) caused by metabolic stress and GI transit changes. This returns to baseline at 8–15 months [32]. They experience a transient acute visceral aversive response followed by adaptation to nausea and satiety driven by incretin reconfiguration [7,41]. This is accompanied by an early weight loss peak and a high rate of T2D remission dependent on incretin dynamics. SG patients experience a moderate acute GDF-15 alteration depending on baseline inflammatory status and comorbidities, which decreases at 12 months [7,32]. They achieve early satiety through mechanical gastric volume restriction and altered gastric emptying with a reduced visceral aversive component. Their metabolic trajectory is influenced by baseline eGFR, renal function, and glycemic/T2D status.

The anorexigenic effect mediated by GDF-15 involves changes in food preferences, delayed gastric emptying through vagal efferents, and the induction of CTA.

Signaling dependent on the GFRAL/RET receptor complex, endocrinely activated under the effect of muscle mitochondrial stress, plays a functional role in the communication axis between muscle and the brain. This signaling pathway leads to an increase in the hypothalamic expression of the corticotropin-releasing hormone gene. Unlike acute stimulation, the chronic activation of the GDF-15–GFRAL axis mediated by mitochondrial stress does not result in the systemic activation of the hypothalamic–pituitary–adrenal (HPA) axis. This signaling governs restrictive anorexia in the diurnal phase and induces anxiety-like behavior [42,43,44].

The absence of an effect from a single GRASP dose on GDF-15-induced anorexia is likely due to the differing timing of pica and anorexia behaviors, along with the GRASP peptide’s half-life. This suggests that at later time points, the antagonist may no longer be present at concentrations sufficient to prevent anorexia [45].

## 4. Gut Microbiota, SCFA Signaling, Recalibration of SCFA–FFAR2/FFAR3 Circuits and GDF-15 Axis’s Post-Bariatric Adaptation

Obesity impairs intestinal motility and absorption, which opposes the restorative effects of bariatric surgery. Microbial imbalance and reduced SCFAs, particularly butyrate, disrupt tight junctions, increase intestinal permeability, and permit lipopolysaccharides to enter the bloodstream, leading to metabolic endotoxemia and systemic inflammation [46].

RYGB induces more predictable homogeneous microbial and metabolic changes compared to SG, which is associated with a more pronounced inter-individual variability [47,48]. Within the first three months after RYGB, the gut microbiome undergoes rapid adaptive changes. These include increased alpha diversity and a shift from the Bacteroidetes 2 to Bacteroidetes 1 enterotype, driven by significant changes in the intestinal microclimate, such as altered luminal pH and bile acid kinetics [49].

**Figure 1:**
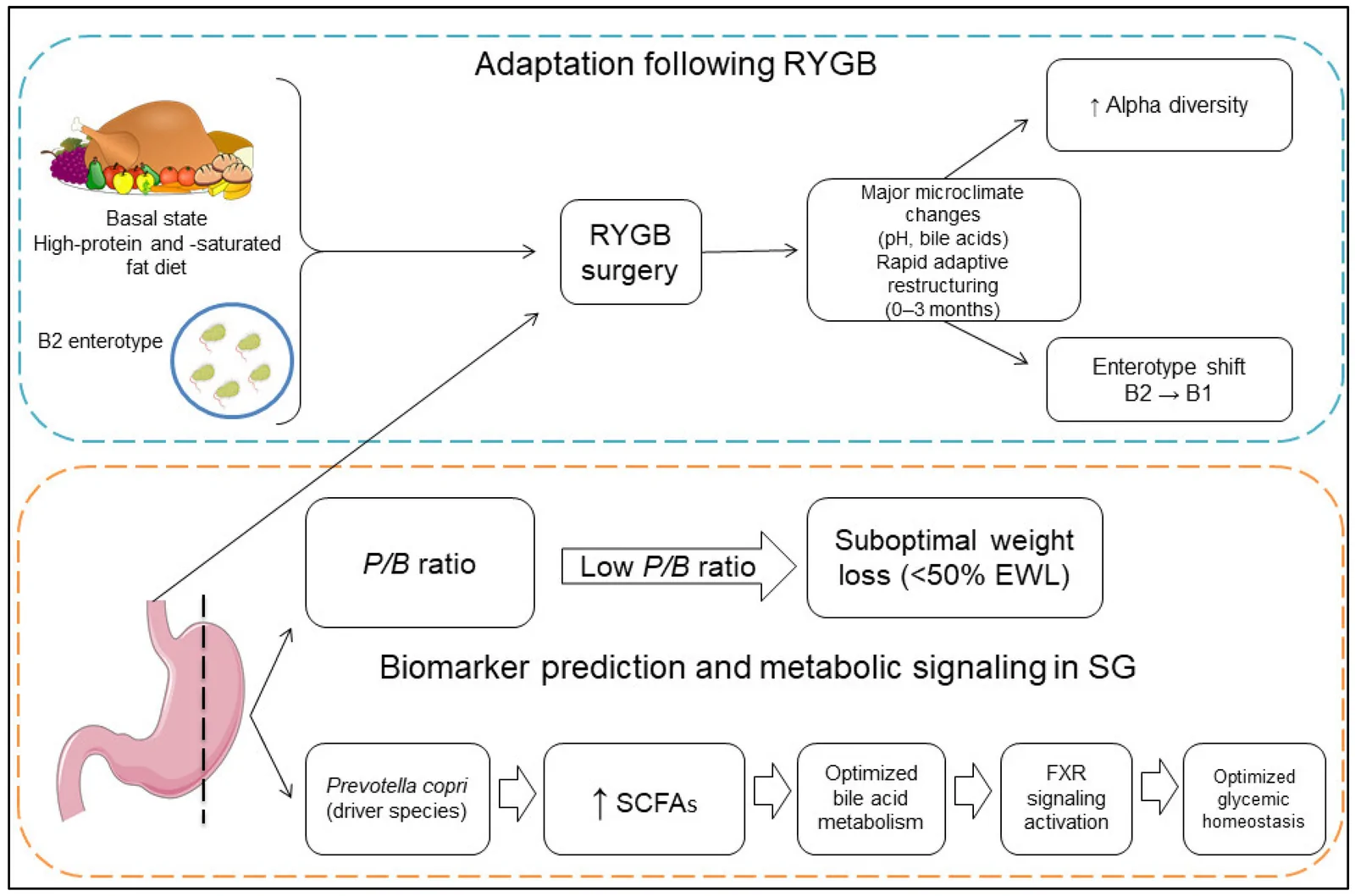
Dual pathways of microbiome remodeling and metabolic signaling following bariatric surgery: RYGB and SG. Abbreviations: EWL, excess weight loss; RYGB, Roux-en-Y gastric bypass; FXR, farnesoid X receptor; SG, sleeve gastrectomy.

A low Prevotella-to-Bacteroides ratio (P/B ratio) predicts suboptimal weight loss success, with excess weight loss below 50% after SG. Experimental models suggest that Prevotella copri supports postoperative glycemic homeostasis through the activation of FXR signaling and normalization of bile acid metabolism [50,51] (Figure 1).

The fermentation of complex carbohydrates and dietary fibers by key bacterial species, including *Roseburia inulinivorans*, *Faecalibacterium prausnitzii*, and *Bacteroides*, produces metabolites such as acetate, propionate, and butyrate, which serve as ligands for G-protein-coupled receptors FFAR2 (GPR43) and FFAR3 (GPR41), highly expressed on enteroendocrine L cells [52,53].

GPR43 is more sensitive to acetate and propionate than to butyrate, while GPR41 is activated by propionate and butyrate. The activation of GPR43 by these metabolites initiates a calcium-dependent signaling cascade that stimulates the secretion of endogenous GLP-1 and PYY. In contrast, GPR41 couples to the inhibitory Gi/o pathway, reducing intracellular cyclic AMP levels [52].

The remission of type 2 diabetes mellitus at 12 months post-surgery is closely correlated with increased bacterial gene abundance and improved functional capacity for anaerobic fermentation. Although the abundance of terminal genes in the butyrate synthesis pathway, *ato* (lysine) and *4hbt* (4-aminobutyrate), increases postoperatively, only the *ato* gene-dependent pathway appears to show a selective and exclusive expansion in patients achieving remission. The preoperative level of *ato* genes appears to be a major predictor of long-term remission at 5 years and correlates positively with the fecal ratio of branched-chain to short-chain fatty acids (BCFA/SCFA) [48].

The remodeling of the gut microbiome and recalibration of metabolic circuits mediated by SCFAs represent a critical point in regulating energy homeostasis [52]. Experimental data suggest a functional convergence between the GDF-15–GFRAL axis and the microbiome–SCFA circuit in regulating food intake, energy homeostasis, inflammation, and intestinal barrier integrity. GDF-15 can remodel the gut microbiota under conditions of tissue stress, while SCFAs and microbiome-derived metabolites modulate endocrine and metabolic signals relevant to appetite control.

Although SCFAs represent major microbial mediators with a suggested role in regulating the intestinal barrier, and GDF-15 is experimentally shown to be an indispensable element for intestinal mucosa protection, the existence of a direct and bidirectional GDF-15–GFRAL-SCFA loop has not yet been convincingly documented [54,55].

A diverse gut microbiome rich in SCFA-producing bacteria reinforces the epithelial barrier by upregulating tight junction proteins such as occludin, zonula occludens-1, and claudins. It limits the systemic translocation of LPS, preventing Toll-like receptor 4 (TLR4)-dependent meta-inflammation and attenuating stress signals that overstimulate GDF-15 synthesis [56,57].

Propionate modulates GLP-1 secretion at the level of enteroendocrine L cells through a direct mechanism, via the allosteric activation of the GPR41 receptor, and indirectly, by stimulating intestinal gluconeogenesis and transmitting the signal through vagal afferents of the gut–brain axis [52]. Although the metabolic actions of the GLP-1 and GDF-15 pathways are complementary, and their concomitant administration as dual agonists shows synergistic and additive therapeutic potential [58], it is not yet clear whether the propionate–GLP-1 postbiotic network can influence endogenous GDF-15 synthesis. Butyrate exerts epigenetic regulation by inhibiting histone deacetylase, altering chromatin accessibility, and upregulating the transcription of the proglucagon gene [52,53].

Song et al. [59] showed that semaglutide monotherapy exacerbates pro-inflammatory intestinal dysbiosis in subjects with obesity by enriching opportunistic pathogens through weakening host immune surveillance via reduced IFN-γ secretion by intraepithelial lymphocytes through the cAMP/PKA pathway and by prolonging gastrointestinal (GI) transit time, which limits carbohydrate availability in the colon, shifting bacterial metabolism from saccharolysis to proteolysis and increasing cecal pH. This shift generates a pro-inflammatory microbial signature that mediates rapid weight regain after stopping treatment, a critical adverse effect that adjuvant dietary fiber supplementation can attenuate.

Understanding how compounds like propionate or butyrate affect GFRAL expression may help restore central sensitivity. Administering targeted postbiotics to post-bariatric patients at a weight loss plateau could replenish GFRAL stores and resensitize the brain to endogenous GDF-15 produced after surgery. Nutritional protocols should have an important place in establishing a holistic intervention that addresses the metabolic, microbiotic, and neuro-affective aspects of obesity.

## 5. Pharmacological Perspectives, Comparative Therapies, and Translational Hypotheses

### 5.1. Comparative Evaluation of Bariatric Surgery, GLP-1 Receptor Agonists, and Exogenous GDF-15

While Jastreboff et al. [60] suggested that the weekly administration of tirzepatide over an active period of 176 weeks results in a substantial mean weight reduction of up to −19.7% for the 15 mg dose, Abdrabou Abouelmagd et al. [61] refined these findings by revealing that the triple agonist retatrutide administered at therapeutic doses of 12 mg can induce a record mean weight reduction of 24.2% after only 48 weeks, with a consistently downward trajectory throughout monitoring.

In accordance with data obtained by Jastreboff et al., the study conducted by Rao et al. [62] reported that the fixed-dose combination CagriSema combines the complementary mechanisms of semaglutide and cagrilintide, an amylin analog that activates central receptors to enhance satiety, leading to dual appetite suppression.

While cagrilintide monotherapy reduces weight and modestly improves blood pressure without significant glycemic impact, both approaches have an acceptable safety profile dominated by GI adverse events, with discontinuation rates comparable to the placebo group.

Furthermore, orforglipron produces a significant reduction in HbA1c from baseline in patients with type 2 diabetes over 40 weeks, while in adults with obesity, the use of this agent resulted in a mean weight loss ranging between −9.4% and −14.7% from baseline, compared to a reduction of −2.3% recorded in the placebo group over 36 weeks [63]. This complex profile indicates the sensitivity of clinical reporting and high real-world discontinuation rates, consolidating a direct therapeutic intersection in the long-term clinical management of obesity (Table 3).

None of these agents have been compared with bariatric surgery in a head-to-head randomized trial, and none have an established perioperative indication; the values shown derive from separate cohorts that differ in duration, population and endpoint definition. The major translational failure of GDF-15 agonists, such as MBL949 and LY3463251, reveals a critical barrier: marked digestive toxicity and extremely modest weight efficacy in humans. While Smith et al. [69] show that the administration of the agonist MBL949 led to limited GI tolerability, recording nausea rates of 70.7% and vomiting rates of 39.0% in the pooled treated group, accompanied by a statistically significant but clinically minor weight loss, Benichou et al. [70] show that the analog LY3463251, a fusion protein obtained by conjugating GDF-15 with a monomeric IgG4-Fc domain, generated modest human weight reductions of only 3% compared to placebo over a 12-week treatment period. The therapeutic efficacy of the molecule at higher doses was limited by GI intolerance manifested as dose-dependent nausea and emesis, despite a prolonged mean half-life of approximately 7.5 days among human subjects.

Treatment withdrawal reveals significant weight regain following therapy discontinuation, pointing to the need for research focused on long-term maintenance strategies [66].

Real-world studies indicate much higher one-year discontinuation rates, ranging from 37% to 81%, justified by insufficient clinical efficacy and global drug supply shortages [71]. For bariatric patients with insufficient weight loss or weight regain after surgery, adjuvant GLP-1 receptor agonists are a clinical option to normalize surgical outcomes, yet because obesity is chronic, discontinuing this long-term pharmacological therapy generally leads to weight regain and the loss of therapeutic benefits [3].

Russo et al. [72] report anatomical changes and a 14.7% rate of postoperative gastroesophageal reflux in bariatric patients. Jastreboff et al. note that stopping tirzepatide leads to a rapid weight regain of about 7% and a reversal of glycemic improvements within 17 weeks [60]. This reliance on ongoing therapy is similar to the tolerability challenges seen with non-incretin modulators such as the GDF-15 axis.

Body composition analyses by Podder et al. [67] show that, on average, approximately 25% to 40% of the total weight lost under potent agonist therapy comes from lean mass. Specifically, DXA assessments documented mean reductions of 6.9 kg of lean soft tissue for semaglutide 2.4 mg, representing approximately 40% of total weight loss in the STEP 1 subgroup, and 5.6 kg of lean soft tissue for tirzepatide, representing approximately 26% of total reduction in the SURMOUNT-1 subgroup. Szafraniec et al. [39] show that bariatric surgery induces a 10–12% loss of fat-free mass (FFM) over 24 months, characterized by aggressive asymmetric changes concentrating 67.4% of the one-year degradation in the first postoperative month.

Dutta et al. [73] showed that adjuvant liraglutide treatment in post-bariatric patients is associated with a statistically decrease in lean mass (−3.01 kg), and muscle mass deterioration remains a clinical concern requiring further evaluation.

The development of certain small molecules was abandoned early due to liver failure, although the oral bioavailability of semaglutide with the SNAC enhancer remains at an extremely low level of 0.4–1% [74].

A well-defined safety profile is observed for the non-peptidic small molecule agonist orforglipron, whose adverse reactions are dominated by GI manifestations, while pharmacokinetic data from early clinical trials reveals the absence of acute hepatocellular or cholestatic toxicity, with no clinically significant increases in transaminases or total bilirubin reported [68].

The current literature supports the protective role of protein intake in preserving FFM and metabolically active muscle [75,76]. However, the effects of a daily protein intake of 1.0–1.5 g/kg of ideal body weight (IBW) on post-bariatric eating behavior, appetite, and satiety have not been thoroughly studied.

While clinical guidelines consider this protein target adequate [77], achieving 1.5 g/kg IBW per day is challenging. Bariatric patients often develop severe aversions and intolerances to protein-rich foods, particularly red meat, making it extremely difficult to reach targets of 1.0–1.5 g/kg/day [40,78]; from a behavioral perspective, bariatric surgery itself causes major reductions in hedonic hunger and binge eating scores [38,79].

Fewer than 10% of patients meet this goal at any postoperative time point, often due to reduced intake, intolerance, low digestibility, or malabsorption. To reach the minimum threshold of 60 g/day, protein supplementation is essential, especially for women in the first postoperative month. However, adherence to supplementation declines within the first year [40,80]. Although fluctuating results in the specialized literature are attributed to different monitoring protocols and the use of protein supplements, sex-differentiated analysis reveals a positive association between higher protein intake and the optimization of postoperative body composition exclusively in men, with a similar impact not being identified in women [80].

Fibroblast Growth Factor 21 (FGF-21) acts as a hormonal sensor for the carbohydrate-to-protein ratio. Protein restriction increases FGF-21 secretion.

In contrast, a CRHP diet reduces circulating FGF-21 levels by up to 54%, regardless of weight loss. This reduction is synergistically associated with the limitation of carbohydrates, which are major stimulators of FGF-21, and with protein supplementation, supporting the role of FGF-21 in regulating macronutrient intake feedback [37].

The current consensus should be viewed through the lens of significant methodological and clinical discrepancies. Some authors indicate that adequate protein intake can reduce the loss of muscle mass. Abiri et al. [75] and Golzarand et al. [76] indicate that an intake of ≥1.2 g/kg/day or ≥60 g/day limits excessive FFM loss. Zhao et al. [81] suggest that whey protein supplementation in post-bariatric patients could attenuate global weight loss and body mass index (BMI) decrease. However, the current literature remains limited by small sample sizes, the methodological heterogeneity of included studies, and a level of clinical certainty evaluated as moderate to very low, with additional large-scale RCTs.

Ben-Porat et al. [77] point out that current guidelines are based on low-quality evidence and that well-controlled studies such as Romeijn et al.’s [82] found no clear advantages of high-protein diets on muscle mass preservation, highlighting the urgency of long-term studies using valid methods to assess body composition and protein status.

A protein intake of up to 1.5 g/kg IBW/day is considered the ideal upper limit for active patients [78]. Exceeding this amount may increase the risk of renal impairment. For patients with pre-existing chronic kidney disease (CKD), protein intake should be limited to 0.8–1.0 g/kg IBW post-bariatric to maintain metabolic safety [83]. Clinical evidence supports a post-bariatric protein intake of 1.2–1.5 g/kg IBW/day to help preserve FFM and improve eating behavior by promoting satiety [38,75].

**Figure 2:**
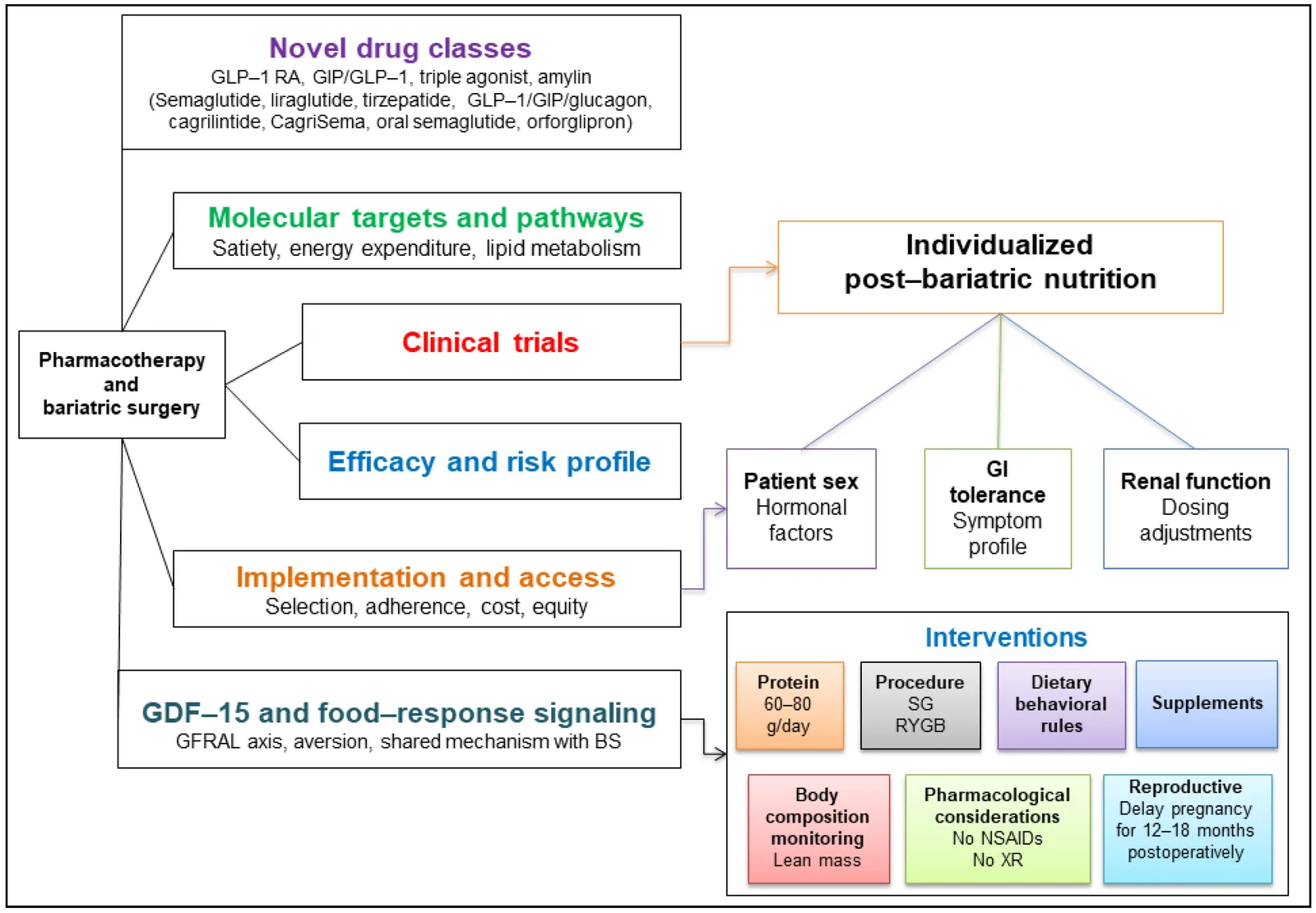
Proposed algorithm integrating pharmacotherapy, bariatric surgery, and postoperative nutrition, structured around clinical stratification and targeted interventions. (Abbreviations: GLP-1 RA, glucagon-like peptide-1 receptor agonist; GIP/GLP-1, glucose-dependent insulinotropic polypeptide/glucagon-like peptide-1; NSAIDs, nonsteroidal anti-inflammatory drugs; XR, extended release.).

Post-bariatric nutritional recommendations should take into consideration individualized needs based on patient sex, digestive tolerance, and renal function, avoiding a universal approach and promoting long-term prospective evaluations (Figure 2).

### 5.2. MT1-MMP Cleavage Axis: Mechanistic Insights and Speculative Biomarker Potential

**Figure 3:**
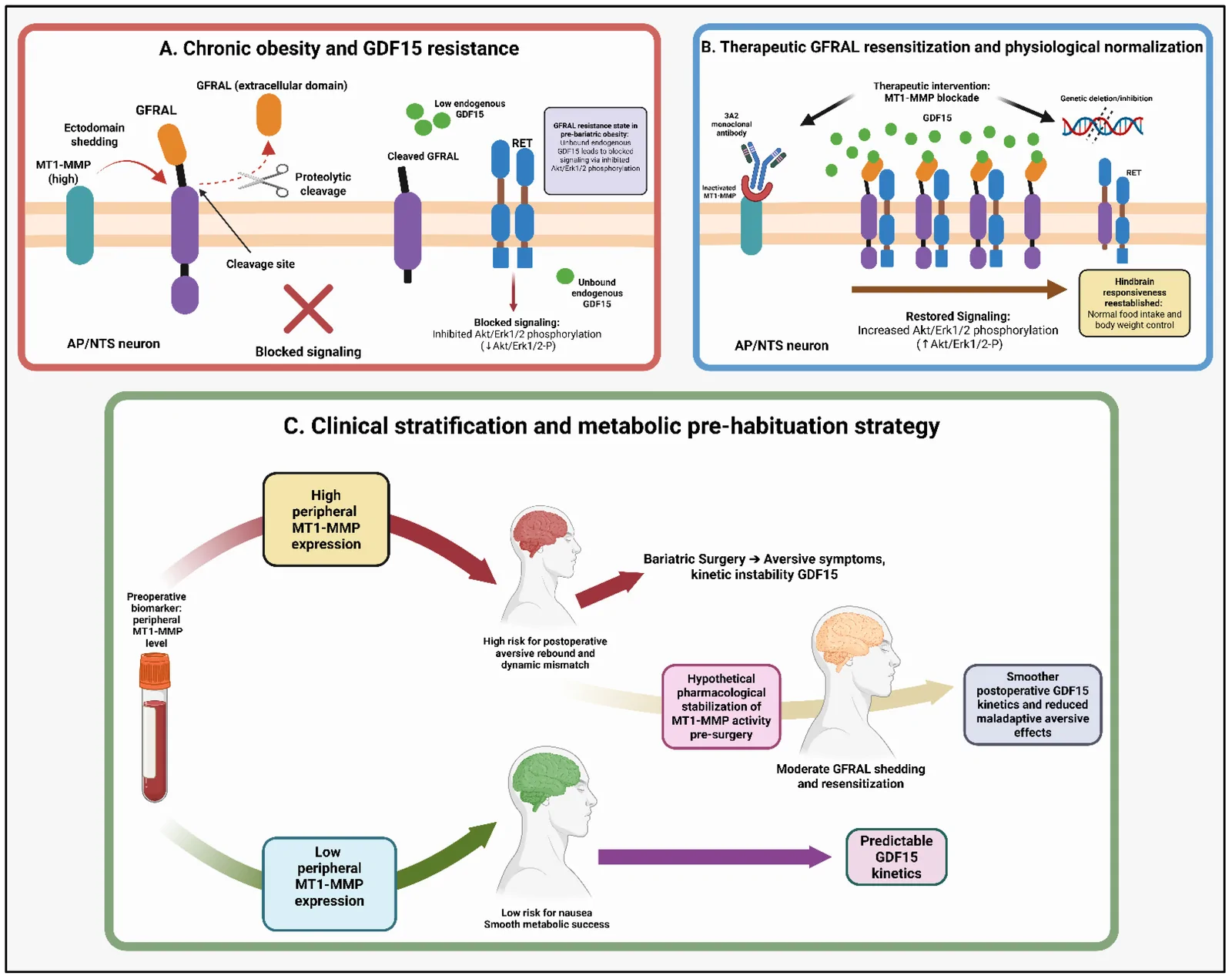
The molecular mechanisms of MT1-MMP-mediated GFRAL cleavage, experimental resensitization in rodents, and a hypothetical translational stratification framework. (**A**) In pre-bariatric obesity, increased MT1-MMP (MMP14) activity at the AP/NTS neuronal membrane cleaves GFRAL at the R333-K site, shedding the ectodomain. The cleaved GFRAL cannot bind endogenous GDF-15, which blocks downstream signaling by inhibiting Akt and Erk1/2 phosphorylation, resulting in GDF-15 resistance. (**B**) Inhibiting or genetically deleting MT1-MMP prevents GFRAL ectodomain shedding. Restoring intact GFRAL/RET receptor complexes enables GDF-15 binding and downstream signaling, increasing Akt and Erk1/2 phosphorylation. This contributes to hindbrain responsiveness, normalizes food intake, and supports body weight control. (**C**) The preoperative measurement of peripheral MT1-MMP levels could enable patient stratification. High MT1-MMP expression could suggest a higher risk of postoperative GDF-15 kinetic instability and aversive symptoms. The pre-surgical proposed pharmacological stabilization of MT1-MMP activity may reduce GFRAL shedding, resensitize the receptor complex, and stabilize GDF-15 kinetics after bariatric surgery. Patients with low MT1-MMP expression could have a lower risk of severe nausea and could have more predictable GDF-15 kinetics. Abbreviations: MT1-MMP, membrane type 1 matrix metalloproteinase; GFRAL, GDNF family receptor alpha-like; AP/NTS, area postrema/nucleus tractus solitarii; RET, rearranged during transfection.

Figure 3 presents a hypothetical model derived from preclinical diet-induced obesity mouse models. In diet-induced obesity mouse models, MT1-MMP expression and neuronal activity increase in the AP and NTS. This activity leads to site-specific GFRAL ectodomain cleavage at R333-K and reduces downstream Akt and Erk1/2 signaling.

MT1-MMP directly interacts with and cleaves the GFRAL receptor at the R333-K site, removing its ectodomain from the neuronal membrane. This process prevents GDF-15 binding and inhibits downstream anorexigenic signaling by reducing Akt and Erk1/2 phosphorylation, which contributes to resistance to endogenous GDF-15 in obesity.

While clinical studies show that metabolic factors such as FGF19 and FGF21 respond differently to weight loss from diet or bariatric surgery, restoring GDF-15 sensitivity by genetically inhibiting MT1-MMP or pharmacologically blocking it with the selective monoclonal antibody 3A2 offers a promising strategy for the non-homeostatic regulation of food intake and body weight.

Unlike in obesity, where the proteolytic cleavage of GFRAL is accelerated, the specific inhibition of MT1-MMP activity achieved experimentally through genetic deletion or pharmacological intervention prevents receptor degradation, restores GFRAL availability on the neuronal membrane surface, and improves hindbrain responsiveness to the anorexigenic signaling of GDF-15.

Although no direct evidence shows GFRAL ectodomain cleavage by MT1-MMP/MMP14 in human tissues, studies in obese mouse models show that MT1-MMP can cleave and inactivate GFRAL, reducing GDF-15–GFRAL signaling. Independent studies also suggest that MT1-MMP regulates membrane receptor availability through ectodomain shedding, affecting receptors such as the insulin and LDL receptors, which impacts metabolic homeostasis, insulin resistance, and lipid metabolism [18,44,84].

If a peripheral index of MT1-MMP activity is validated as a predictor of adverse risk, clinicians may consider metabolic pre-habituation strategies for high-risk patients.

Stabilizing MT1-MMP activity before surgery could help regulate GFRAL shedding and resensitization, potentially improving postoperative GDF-15 kinetics and reducing maladaptive aversive effects.

This approach remains hypothetical, and effective pharmacological stabilization methods in humans have not yet been established.

This proposed format aims to connect established rodent proteolytic pathways with a clinical framework, proposing preoperative peripheral MT1-MMP activity as a potential biomarker to identify patients at risk for postoperative GDF-15 instability, severe nausea, and adverse dietary symptoms.

The model is a conceptual framework that requires empirical validation. There is no direct evidence of GFRAL ectodomain cleavage by MT1-MMP in human tissues. Applying these preclinical neurometabolic mechanisms to patients undergoing RYGB or SG remains speculative.

We hypothesize that quantifying a peripheral index of MT1-MMP activity alongside GDF-15 kinetics could establish a non-invasive biomarker framework for perioperative risk stratification. The peripheral venous blood sampling of soluble MT1-MMP cleavage fragments and circulating GDF-15 offers high practical feasibility and minimal patient burden compared to central hindbrain or invasive tissue sampling.

Current standard-of-care preoperative evaluations rely primarily on basic liver and renal panels or generic psychometric questionnaires, which fail to capture real-time neurometabolic flux or post-surgical factor dynamics. Implementing routine multiplex immunoassay or ELISA platforms presents a cost-effective addition to standard laboratory workups.

Under this prospective hypothesis, elevated preoperative peripheral MT1-MMP activity is proposed to correlate with higher risk for postoperative GDF-15 kinetic instability, severe nausea, food aversion, and postprandial intolerance. Identifying high-risk RYGB or SG candidates could enable targeted metabolic pre-habituation strategies, including pre-surgical MT1-MMP stabilization or nutraceutical modulation via bioactive compounds such as resveratrol.

This model includes several potential failure points and technical limitations that require transparent evaluation. First, peripheral MT1-MMP expression drives systemic tissue remodeling, matrix degradation, and inflammatory pathways, which may generate substantial off-target background noise in serum and confound peripheral biomarker accuracy. Second, humans have not established a direct quantitative correlation between circulating MT1-MMP cleavage fragments and central GFRAL receptor status in the AP/NTS. Furthermore, pharmacological methods for targeted MT1-MMP stabilization in human clinical settings remain unvalidated.

The integrated profiling of peripheral MT1-MMP, GDF-15 dynamics, and bioactive nutraceutical responses remains an unproven research model. Rigorous pilot clinical trials and translational animal models are strictly required to validate whether peripheral MT1-MMP metrics can reliably predict post-bariatric vulnerability phenotypes and severe aversive outcomes. Further integrated research on MT1-MMP, GDF-15, and nutraceutical profiles in bariatric patients is needed to identify vulnerability phenotypes related to food aversion, postprandial intolerance, or persistent dietary discomfort, using pilot clinical studies and animal models.

### 5.3. Postoperative Neurobehavioral Outcomes: Competing Explanations

Postoperative neuropsychiatric outcomes are multifactorial, and several explanations carry stronger human evidence than GDF-15 signaling. They are not mutually exclusive, but they are rarely disentangled in the same cohort [12,15].

Psychiatric morbidity is already high at the time of candidacy, with pooled preoperative prevalences of 19% for depression and 17% for binge eating disorder [85]. Depressive symptoms improve on average after surgery [85,86], with one subgroup deteriorating, and umbrella-level evidence indicates an increased risk of self-harm and suicide, with alcohol use disorder risk rising particularly from the third postoperative year [87,88]. Postoperative psychiatric symptoms may therefore reflect the trajectory of pre-existing disease, compounded by unmet expectations regarding weight or appearance, altered family and social dynamics, and the loss of food as a coping strategy, rather than a surgically induced state.

Nutritional deficiency is a second, well-documented pathway. Post-bariatric declines in albumin, vitamin B12 and zinc are common, and thiamine deficiency has been reported to cause Wernicke encephalopathy in the context of severely reduced intake, including under concomitant GLP-1 receptor agonist therapy [89]. The reduced postoperative absorption of antidepressants may further destabilize previously controlled psychiatric illness [90].

Third, the increased risk of alcohol use disorder after bariatric surgery has a parsimonious pharmacokinetic explanation. In a 3-year prospective study, both RYGB and SG approximately doubled peak ethanol concentration and total systemic exposure and halved time to peak (9–15 min postoperatively versus 25–29 min preoperatively), with more pronounced changes after gastric bypass [91]. Faster and higher alcohol exposure increases reinforcing effects without requiring any change in central reward signaling and accounts for much of what is described as addiction transfer.

Against this background, it is not clinically established whether or how perioperative MT1-MMP activity is linked to postoperative psychological or psychiatric outcomes. Attributing these outcomes to the GDF-15/GFRAL/RET axis therefore requires designs that measure the axis and the alternatives above concurrently.

## 6. Knowledge Gaps, Research Priorities and Translational Challenges

Although circulating GDF-15 rises postoperatively after RYGB and SG, with 12-month postoperative elevations predicting total weight loss, the precise perioperative kinetic trajectories and comparative secretion dynamics between RYGB and SG remain insufficiently characterized in primary longitudinal cohorts [92,93,94].

The variability in circulating GDF-15 levels following metabolic surgery remains considerable and lacks standardization across the current literature. This heterogeneity may reflect differences in intervention type, the timing of assessment, the degree of hepatic comorbidity improvement, and changes in concomitant pharmacological therapies. Di Vincenzo et al. [30] report a divergent effect in bariatric patients, showing that preoperative metabolic syndrome attenuates the adaptive postoperative secretory response of GDF-15. A critical aspect is the lack of validated concentration thresholds distinguishing homeostatic satiety signaling from pathological or pharmacological surges [92,93]. Baseline circulating GDF-15 under physiological conditions ranges from approximately 200 to 1200 pg/mL (0.2–1.2 ng/mL). Pathological or pharmacological levels above 1500 pg/mL activate the hindbrain GFRAL–RET receptor complex [92,93,95,96]. This binary assembly recruits aversive projections to the parabrachial nucleus, inducing dose-limiting emesis, visceral malaise, and CTA [92,93,97].

The relationship between persistent GDF-15 elevation, visceral discomfort or aversion, and postoperative psychiatric symptoms remains unestablished [14,15,16,17]. To our knowledge, no cohort has combined serial perioperative GDF-15 measurement with standardized psychiatric assessment in the same patients, and preclinical models dissociating surgical from dietary weight loss provide no behavioral readout [36]. Prospective studies pairing early postoperative GDF-15 sampling with validated instruments for anhedonia, depressive symptoms and substance use are required, with prespecified adjustment for pre-existing psychiatric disease, micronutrient status and altered post-RYGB alcohol pharmacokinetics. Until then, a protective effect of GDF-15/GFRAL/RET modulation on reward circuitry remains an untested hypothesis. A parallel gap concerns skeletal muscle, where no integrated evaluation links bariatric surgery, GDF-15/GFRAL dynamics, and postoperative changes in muscle mass, strength and quality, despite the characterization of GDF-15 as a myomitokine [17]. Furthermore, the current literature lacks an integrated evaluation linking bariatric surgery, GDF-15/GFRAL dynamics, and the molecular mechanisms driving postoperative changes in skeletal muscle mass, strength, and quality. This lack of direct empirical evidence underscores the need for translational studies to explain the crosstalk between central GDF-15 signaling, adipocyte remodeling, and post-bariatric muscle degradation.

The MT1-MMP cleavage of GFRAL has been proposed to reduce central receptor abundance and blunt GDF-15 signaling, offering a theoretical explanation for peripheral GDF-15 resistance in chronic metabolic disease. However, its diagnostic utility and role as a circulating peripheral biomarker in bariatric candidates remain speculative and unvalidated in human cohorts [92]. Whether MT1-MMP (MMP14) can serve as a biomarker for stratifying the risk of exaggerated aversive responses after bariatric surgery first requires identifying a measurable surrogate, discussed below (Figure 1).

To date, no direct evidence links nutraceuticals, micronutrients, or phytochemicals to preoperative MMP14 expression. Future integrated investigations of MT1-MMP, GDF-15, and nutraceutical profiles in bariatric research are urgently needed to identify vulnerability phenotypes related to food aversion, postprandial intolerance, or persistent dietary discomfort using pilot clinical studies and animal models.

Significant methodological limitations in clinical studies constrain progress in evaluating perioperative GDF-15 dynamics. Existing surgical cohorts often have small sample sizes and short observational windows that capture only the first 3 postoperative days instead of standardized, extended longitudinal sampling. They also include heterogeneous control groups [98]. Interpreting post-bariatric GDF-15 trajectories requires the rigorous control of major clinical confounders [92,98]. Perioperative micronutrient alterations must be systematically accounted for [94,98,99].

Pre-existing psychiatric conditions such as depression or binge eating disorder, psychosocial factors, and altered post-RYGB alcohol pharmacokinetics or substance use are essential unmeasured variables that can alter appetite and weight trajectories. This necessitates strict prospective standardization [99]. Comprehensive research is also needed to clarify the relationship between GDF-15 and the microbiome and to determine whether specific targeted compounds can recalibrate the GDF-15/GFRAL/RET axis in the context of dysbiosis and metabolic inflammation.

Before GDF-15/GFRAL-targeted perioperative interventions enter clinical practice, prospective multicenter human clinical trials are required to establish safety, efficacy, and therapeutic durability [92,93,98]. The primary translational bottleneck of exogenous GDF-15 agonism is its narrow therapeutic index because biological efficacy in appetite suppression overlaps with dose-limiting nausea and emesis [92,97]. To widen this therapeutic window, future studies must evaluate dose-sparing co-formulation strategies, exposure-shaping titration ramps, and biased agonists engineered to favor anorectic ERK1/2 signaling over aversive PLC-γ cascades [92,97].

Phytochemicals that activate ISR/ATF4, p53, or NRF2 have been extensively studied in metabolic contexts, but their effects on the GDF-15/GFRAL/RET axis remain underexplored. These compounds may be promising candidates for future research. Studies should assess whether they can modulate the GDF-15 axis in relation to dysbiosis, microbiome-associated metabolic disorders, and inflammatory intestinal phenotypes seen in obesity and metabolic syndrome. Investigating how phytochemicals influence the GDF-15/GFRAL/RET axis, either directly or via the microbiome, may advance personalized medicine. Polyphenols from pomegranate, green tea, cocoa, soy, and red ginseng should be evaluated as potential modulators that fine-tune axis activity without excessive agonism, especially in translational studies. Integrating these studies with risk stratification and predictive modeling can help identify patient subgroups most likely to benefit from GDF-15 modulation.

Ultimately, clinical translation depends on developing explicit patient stratification criteria. Baseline endogenous GDF-15 concentrations should phenotype candidates by distinguishing individuals with intact, responsive signaling from those with pathway saturation or resistance [92]. Stratification protocols must also include age- and sex-specific diagnostic cut-offs [93], adjust for the estimated glomerular filtration rate (eGFR) due to renal clearance [92,100], and screen rigorously for concurrent metformin or incretin-based therapies.

## 7. Conclusions

The GDF-15/GFRAL/RET axis is a candidate neurometabolic pathway linking the metabolic adaptation that follows bariatric surgery to its postoperative behavioral consequences. Its contribution to metabolic outcomes is supported by human kinetic data; its contribution to psychiatric outcomes is not and remains hypothetical. The perioperative vulnerability windows defined in Section 3.2 provide the temporal frame within which these questions can be tested; the acute window remains entirely unsampled in humans.

In the postoperative period, multiorgan stress may sustain supraphysiological GDF-15 levels, potentially converting this homeostatic signal into a chronic activator of aversive circuits by engaging neuroanatomical networks.

Whether persistent activation contributes to a phenotype of anxiety, anhedonia and sickness-like behavior beyond appetite suppression has not been tested in humans. The alternative mechanisms discussed in Section 5.3—pre-existing psychiatric disease, micronutrient deficiency, the altered absorption of psychotropic medication, and procedure-related changes in alcohol pharmacokinetics—currently account for these outcomes more parsimoniously.

The pleiotropic effects of GDF-15 and its interactions with other post-bariatric mediators, such as GLP-1, PYY, and the gut microbiome, complicate the interpretation of this axis’s specific effects on adult behavior and neural plasticity. Future research should consider nutraceutical interventions, including those that modulate the microbiome, which appear linked to circulating GDF-15 levels.

Future research should prioritize human interventional studies that isolate interdependent variables to determine whether the GDF-15/GFRAL/RET axis represents an independent and viable therapeutic target in this context. A detailed examination of its interactions with other natural compounds is warranted. Whether the longitudinal monitoring of this biomarker, combined with selective GFRAL modulation, could influence neurobehavioral outcomes without compromising metabolic remission is an open question that current data cannot answer.

Evaluating the long-term cost–benefit ratio of GLP-1-based therapies, used as a non-invasive alternative or a synergistic post-bariatric strategy, requires carefully weighing metabolic remission against systemic risks. Optimizing clinical outcomes and reducing long-term adverse effects require a precision medicine approach rigorously stratified by age, sex, baseline nutritional status, bariatric procedure type, enterotype, and individual dietary pattern. This personalized approach should be seen as a way to maximize therapeutic durability while minimizing sequential metabolic and psychiatric risks.

## Figures and Tables

**Table 1:** Interspecies synthesis of GDF-15/GFRAL mechanisms, anatomical substrates, and their relevance to BS (RYGB/SG).

Author, Year, Reference	Experimental Model (Species, Group, Design)	Primary Biological Mechanism	Anatomical and Molecular Substrate	Relevance to the RYGB/SG Context
Borner et al., 2020 [13]	Multispecies in vivo: C57BL/6J mice (cisplatin). Lean and obese SD rats (GDF-15 i.c.v./i.p.). Suncus murinus shrews (GDF-15 i.p.).	GDF-15 induces emesis (shrew) and pica + taste aversion (rodents). Pica precedes anorexia at all doses; identical in obese animals. Ondansetron does not prevent pica → independent of 5-HT3R.	GFRAL–RET complex, restricted to AP/NTS. c-Fos in GFRAL+ neurons from AP/NTS (mouse). In shrew: c-Fos in NTS, not in AP/DMV → interspecies difference. Probable peripheral source: liver.	Increased GDF-15 acts through a pathway that obligatorily couples anorexia with nausea. Explains food intolerance and food aversions post-RYGB/SG.
Worth et al., 2020 [22]	C57BL/6J mice and Cre-reporter lines (*n* = 4–12/group). SD rats for pica. RNAscope and retrograde tracing (*n* = 3).	GDF-15 → anorexia, taste and place aversion, pica. Ablation of CCK neurons or devazepide attenuates anorexia. Anti-GFRAL antibody completely blocks GDF-15 and cisplatin effects; alone it has no effect.	GFRAL on a subpopulation of glutamatergic CCK neurons in the AP and medial NTS (60%/31% colocalization). Direct projections to lPBN (partially CGRP+) → PVH, CeA, ovBNST. GFRAL absent in the intestine and vagus nerve.	Defines the complete circuit through which GI GDF-15 can act centrally. The pathway does not participate in physiological satiety—it is activated only when GDF-15 increases.
Zhang et al., 2021 [23]	Mice; AP atlas via sNuc-seq (3678 cells). Cre lines, chemogenetics, optogenetics, ablation, knockout/rescue *Glp1r*. Behavioral tests *n* = 4–18/group.	Activation of GFRAL+ or SLC6A2+ neurons induces aversion; CALCR+ does not. Ablation of GFRAL+ abolishes aversion to LiCl and LPS. Stimulation also produces cardiovascular and gastric motility changes.	AP: only 4 types of excitatory neurons, 3 inhibitory. GFRAL marks excitatory cluster 4, which co-expresses *Glp1r* and *Casr*. Long projections exclusively excitatory, dense over CGRP+ neurons in PBN.	GFRAL and *GLP1R* are located on the same neurons—convergence node of post-bariatric signals. Possible synergistic action of GDF-15/GLP-1 and explanation for gastric motility changes.
Dolo et al., 2022 [24]	Actual BS. Obese SD rats, *N* = 40; 30 made diabetic with STZ, then RYGB (10), SG (10), SHAM (10); 10 normal control. 8-week follow-up.	Weight loss: 18.5% (RYGB), 15.2% (SG). Intake reduction: 37.6% (RYGB) vs. 26.9% (SG). Glucose tolerance and HOMA-IR improved; GLP-1 increased, more after RYGB. SHAM: no changes.	Plasma GDF-15 almost doubled at 4 weeks, maintained at 8. Increased tissue expression in pouch, jejunum, and ileum after RYGB; after SG only in ileum. Decreased hepatic expression after RYGB. Increase occurs along the alimentary tract, not in excluded loops.	The only direct evidence that RYGB and SG increase circulating and tissue GDF-15. Sustained nature (8 weeks) + SHAM group rule out simple surgical stress. Limitation: descriptive study, without nausea assessment.

Abbreviations: AP—area postrema; NTS—nucleus of the solitary tract; DMV—dorsal motor nucleus of the vagus; PBN/lPBN—parabrachial nucleus/lateral; CeA—central nucleus of the amygdala; PVH—paraventricular nucleus of the hypothalamus; ovBNST—oval subnucleus of the bed nucleus of the stria terminalis; CGRP—calcitonin gene-related peptide; CCK—cholecystokinin; GDF-15—growth differentiation factor 15 (MIC-1); GFRAL—GDNF family receptor alpha-like; RET—rearranged during transfection; GLP-1/GLP1R—glucagon-like peptide 1/its receptor; Casr—calcium-sensing receptor; 5-HT3R—serotonin receptor 3; RYGB—Roux-en-Y gastric bypass; SG—sleeve gastrectomy; SHAM—sham operation; SD—Sprague-Dawley; STZ—streptozotocin; HOMA-IR—homeostatic model assessment for insulin resistance; sNuc-seq—single-nucleus RNA sequencing; LiCl—lithium chloride; LPS—lipopolysaccharide; i.p.—intraperitoneal; i.c.v.—intracerebroventricular. Note: The paper by Borner et al. is presented in a single row, although it includes four distinct animal models. Values are rendered as reported in the original sources.

**Table 2:** Human clinical evidence on perioperative GDF-15 kinetics, metabolic correlates, and methodological heterogeneity across bariatric procedures.

Author, Year, Reference	Surgical Procedure	Cohort Size (N) and Comparator Group	Sampling Timeline	GDF-15 Kinetic Dynamics	Clinical and Metabolic Associations	Methodological Limitations
Dolo et al., 2020 [33]	SG	*n* = 21 (15 obesity, 6 T2D); no comparator group (preoperative self-control)	Baseline and 1, 3 and 12 months	Increase vs. baseline at 1 month (215.1 → 301.9 pg/mL; *p* < 0.05), peak at 3 months (338.9; *p* < 0.01), sustained at 12 months (329.4; *p* < 0.05).	Only significant correlation: ΔGDF-15 at 3 months with BMI reduction (r^2^ = 0.204). No correlation with ΔFPG or ΔHOMA-IR.	Small, single-center sample; no unoperated control; no sampling within the first 72 h; subgroups unbalanced for age; low baseline values (Cloud-Clone ELISA)
Martinussen et al., 2021 [34]	RYGB and SG	Operated *n* = 26 (10 RYGB + 10 SG; 6 RYGB) vs. matched unoperated controls (*n* = 10 and *n* = 6); post hoc analysis of 3 crossover studies	Cross-sectional, single time point ≥ 12 months after surgery; fasting and postprandial (4–6 h); no preoperative samples	No between-group differences in fasting levels (SG 477, RYGB 513, control 528 pg/mL) and no postprandial response. The abstract reports different values (RYGB 540, control 590).	Association with weight loss cannot be assessed (no preoperative data); GDF-15 correlated with age and with BMI only in controls.	Cross-sectional design, no pre-/postoperative data in the same subjects; 6–10 subjects/group; residual samples stored at −20 °C; patients with diabetes excluded
Salman et al., 2021 [35]	SG	SG *n* = 68 vs. conservative treatment *n* = 58; groups differed at baseline (age, BMI, HOMA-IR)	Baseline and 12 months in both groups; no intermediate time points	SG 409.9 → 699.9 pg/mL (Δ +290) vs. conservative 385.9 → 559 (Δ +173); *p* < 0.001 for the difference between increases.	In SG, ΔGDF-15 positively correlated with weight loss (r = 0.535; independent predictor) and weakly with ΔHOMA-IR and ΔALT (whole cohort). The abstract reports r = −0.793, discordant with the tables.	Non-randomized, single-center; groups imbalanced at baseline; no sampling within the first 6 months; antidiabetic medication not controlled; low baseline values (Cloud-Clone ELISA)
Schmid et al., 2022 [36]	RYGB vs. low-calorie diet (LCD), non-randomized comparison	RYGB *n* = 79 vs. LCD *n* = 81 (baseline BMI 51.7 vs. 43.6 kg/m^2^)	Baseline and 3, 6 and 12 months (GDF-15); an additional sample was collected 3–4 days after surgery in the RYGB subgroup, with no GDF-15 results reported	Decrease at 12 months: LCD 378 → 295 pg/mL; RYGB 481 → 319 pg/mL; no difference between groups. Transient increase at 3 months in patients with advanced fibrosis.	Baseline GDF-15 correlated with HbA1c (r = 0.43; BMI-independent) and NFS (r = 0.39). ROC: advanced fibrosis AUC 0.910; T2D AUC 0.78 (RYGB 0.875); hypertension ≈ 0.7.	Observational, single-center, non-randomized; BMI differed between groups; no lean controls; RYGB only (no SG); fibrosis estimated non-invasively; ROC cut-offs not externally validated; 12-month follow-up
Chaiyasoot et al., 2023 [31]	RYGB and SG (proportion not specified)	Surgery *n* = 41 vs. LEI alone *n* = 41, topiramate *n* = 46, liraglutide *n* = 31, orlistat *n* = 12; lean controls *n* = 46 (baseline only)	Baseline and 1 year; no intermediate time points	Surgery: median decreased 1165 → 606 pg/mL (*p* = 0.003). Comparators: decrease with topiramate, increase with LEI alone (676 → 817), no change with liraglutide/orlistat.	Greater GDF-15 decrease associated with greater weight loss (β = −4.49; *p* = 0.027) in the pooled cohort (*n* = 171), not surgery-specific. Baseline GDF-15 did not predict weight loss.	No intermediate data; RYGB and SG not analyzed separately; single-center, non-randomized; association derived from the mixed cohort; multiplex assay (not ELISA), high baseline levels; Asian population
Di Vincenzo et al., 2025 [30]	SG	*n* = 36 (MetS *n* = 21; no MetS *n* = 15); no unoperated control	Preoperative and postoperative; interval not specified	Baseline: MetS 1360 vs. no MetS 756 pg/mL (*p* = 0.002). After SG: increase only in no MetS (756 → 843; *p* = 0.04); MetS unchanged.	Baseline GDF-15 correlated with glucose (MetS) and insulin (no MetS), associations lost postoperatively; no correlation with weight loss or anthropometry.	Small, single-center sample, Caucasian population; post-SG interval not specified; no unoperated control; no adjustment for medication; brief report
Urones et al., 2025 [7]	SG and RYGB (clinical allocation, non-randomized)	Operated: *n* = 144 at T0, 64/71/106 at 3/6/12 months (12 months: 51 SG + 55 RYGB); normal-weight controls *n* = 72	Baseline and 3, 6 and 12 months	Decrease at 12 months (paired, *n* = 106: 904 → 654 pg/mL; *p* = 0.001), driven by the T2D and hypertension subgroups; SG and RYGB similar; remains above controls.	ΔGDF-15 (T12 − T0, negative) correlated with %EWL (rho = 0.21): smaller decrease = greater %EWL; absolute levels not correlated with weight loss. Hypertension was the only independent predictor.	Varying number of patients over time (144 → 106); heterogeneous comorbidities and medication (metformin); no unoperated controls with obesity; diet and exercise not assessed
Salmón-Gómez et al., 2025 [32]	RYGB	*n* = 104 (normoglycemic *n* = 52; IGT/T2D *n* = 52); no unoperated control	Baseline and a single postoperative time point at 5–15 months	Increase: normoglycemic 482 → 582 pg/mL (*p* < 0.01); IGT/T2D 512 → 575 (*p* < 0.05). Significant only at ≤8 months, not at >8 months.	No correlation with %EWL or %FATLOSS; GDF-15 positively correlated with HOMA-IR, pre- and postoperatively (r = 0.44/0.40).	Single postoperative point within a wide window (time analysis cross-sectional); no unoperated control; White population, single-center, moderate N; IGT and T2D combined; diet not assessed

Abbreviations: %EWL, percentage of excess weight loss; %FATLOSS, percentage of fat mass loss; ALT, alanine aminotransferase; AUC, area under the ROC curve; BMI, body mass index; FPG, fasting plasma glucose; HbA1c, glycated hemoglobin; HOMA-IR, homeostatic model assessment of insulin resistance; IGT, impaired glucose tolerance; LCD, low-calorie diet; LEI, lifestyle education intervention; MetS, metabolic syndrome; NFS, NAFLD fibrosis score; RYGB, Roux-en-Y gastric bypass; SG, sleeve gastrectomy; T2D, type 2 diabetes mellitus; Δ, change (postoperative − preoperative).

**Table 3:** Next-generation anti-obesity pharmacotherapies relative to bariatric surgery (abbreviations: TWL, total weight loss; GLP-1, glucagon-like peptide-1; HbA1c, glycated hemoglobin A1c; CV, cardiovascular).

Medication	Mechanism	Efficacy	Development Status and Position Relative to Bariatric Surgery	Reference
Semaglutide	Reduces appetite Improves glycemic control Cardioprotective effects	15–17% mean TWL (Phase 3)	Approved. Prevents post-surgical regain or optimizes suboptimal response (+4.36–7.83 kg loss); long-term bariatric surgery remains superior by ~9–10% TWL.	[3,64,65]
Tirzepatide	Stronger metabolic and weight loss effects than GLP-1 alone	Up to 22.5% mean TWL (Phase 3); superior to semaglutide (weight and HbA1c)	Approved. Largest weight loss among approved agents, but no head-to-head randomized comparison with bariatric surgery; equivalence not demonstrated.	[3,65,66]
Retatrutide	Suppresses appetite; increases energy expenditure; improves adipose metabolism and hepatic lipid oxidation	Superior efficacy compared to tirzepatide (Phase 3)	Phase 3 ongoing. Late-phase weight loss approaches values reported after bariatric surgery in separate cohorts, without direct comparison.	[3,67]
CagriSema (GLP-1/Amylin Receptor Agonist Combination)	Enhances satiety and energy balance	Early data suggest weight loss ≥ tirzepatide (Phase 3 ongoing)	Phase 3 ongoing. No comparison with bariatric surgery reported.	[3]
Orforglipron	Designed for oral dosing to improve adherence	Reductions in HbA1c, body weight, and CV biomarkers (Phase 1–3)	Phase 3. Oral administration; no comparison with bariatric surgery reported.	[68]
MBL949 (GDF-15 Receptor Agonist)	Signals via GFRAL-RET in the hindbrain	Weight loss at high doses (Phase 1–2) Minimal effect (Phase 2, 14 weeks)	Phase 1–2. Weight loss only at high doses, with nausea in 70.7% and vomiting in 39.0%; no perioperative application established.	[30,32,69]
Bimagrumab	Preserves lean mass during weight loss	Promising data for conserving muscle/lean mass (early phase)	Early phase. Lean mass preservation not yet demonstrated in bariatric populations.	[3,39]

## Data Availability

No new data were created or analyzed in this study. Data sharing is not applicable.

## Abbreviations

The following abbreviations are used in this manuscript:

AP/NTS	Area postrema and the nucleus of the solitary tract
BCFA	Branched-chain fatty acid
BS	Bariatric surgery
CTA	Conditioned taste aversion
FFAR2	Free fatty acid receptor 2
FFAR3	Free fatty acid receptor 3
GDF-15	Growth differentiation factor 15
GDNF	Glial cell line-derived neurotrophic factor
GFRAL	GDNF family receptor alpha-like
GI	Gastrointestinal
GLP-1	Glucagon-like peptide-1
MMP14	Matrix metalloproteinase-14 (alt. name for MT1-MMP)
MT1-MMP	Membrane-type 1 matrix metalloproteinase
PLC	Phospholipase C
RA	Receptor agonist
RET	Rearranged during transfection (receptor tyrosine kinase)
RYGB	Roux-en-Y gastric bypass
SCFA	Short-chain fatty acid
SG	Sleeve gastrectomy
TGF-β	Transforming growth factor beta
TLR4	Toll-like receptor 4
